# Association of Anticancer Immune Checkpoint Inhibitors With Patient-Reported Outcomes Assessed in Randomized Clinical Trials: A Systematic Review and Meta-analysis

**DOI:** 10.1001/jamanetworkopen.2022.26252

**Published:** 2022-08-16

**Authors:** Laura Pala, Isabella Sala, Chiara Oriecuia, Tommaso De Pas, Paola Queirolo, Claudia Specchia, Emilia Cocorocchio, Pierfrancesco Ferrucci, Damiano Patanè, Maristella Saponara, Elisabetta Pennacchioli, Sara Coppola, Giuseppe Viale, Giuseppe Giaccone, Richard D. Gelber, Vincenzo Bagnardi, Fabio Conforti

**Affiliations:** 1 Division of Melanoma, Sarcomas, and Rare Tumors, European Institute of Oncology, Milan, Italy; 2 Oncology Unit, Humanitas Gavazzeni, Bergamo, Italy; 3 Department of Statistics and Quantitative Methods, University of Milan-Bicocca, Milan, Italy; 4 Department of Molecular and Translational Medicine, University of Brescia, Brescia, Italy; 5 Department of Medical and Surgical Specialties, Radiological Sciences, and Public Health, University of Brescia, Brescia, Italy; 6 Division of Medical Oncology, European Institute of Oncology, Istituto di Ricovero e Cura a Carattere Scientifico, Milan, Italy; 7 Department of Experimental Oncology, European Institute of Oncology, Istituto di Ricovero e Cura a Carattere Scientifico, Milan, Italy; 8 Melanoma, Sarcoma, and Rare Tumors Surgery Division, European Institute of Oncology, Istituto di Ricovero e Cura a Carattere Scientifico, Milan, Italy; 9 Department of Pathology, European Institute of Oncology, Milan, Italy; 10 Department of Oncology and Hematology, University of Milan, Milan, Italy; 11 Department of Oncology, Weill Cornel Medicine, New York, New York; 12 Department of Data Science, Dana-Farber Cancer Institute, Harvard Medical School, Harvard T.H. Chan School of Public Health, Boston, Massachusetts; 13 Frontier Science & Technology Research Foundation, Boston, Massachusetts

## Abstract

**Importance:**

The association of immune checkpoint inhibitors (ICIs) with patient quality of life has been poorly explored.

**Objective:**

To evaluate patient-reported outcomes (PROs) assessed in randomized clinical trials (RCTs) of immunotherapy-based treatments.

**Data Sources:**

This systematic review and random-effects meta-analysis used RCTs identified in PubMed, MEDLINE, Embase, and Scopus from database inception to June 1, 2021.

**Study Selection:**

A total of 2259 RCTs were identified that assessed ICIs as monotherapy or in combination with chemotherapy or combined with another ICI and/or targeted therapy vs control groups not containing immunotherapy in patients with advanced solid tumors. Studies were reviewed independently by 2 authors.

**Data Extraction and Synthesis:**

This meta-analysis followed the PRISMA guidelines and recommendations of the Setting International Standards in Analyzing Patient-Reported Outcomes and Quality of Life Endpoints Data Consortium.

**Main Outcomes and Measures:**

The coprimary aims of the meta-analysis were (1) pooled differences between treatment groups in the mean change of PRO score from baseline to 12 and 24 weeks of follow-up and (2) pooled differences between treatment groups in the time to deterioration of PRO score. For each end point, RCTs have been analyzed according to the type of treatment administered in the experimental group: ICIs given as monotherapy, ICIs combined with chemotherapy, or ICIs in association with another ICI and/or with targeted therapies.

**Results:**

Of the 2259 identified RCTs, 34 (18 709 patients) met the selection criteria and were analyzed. In the group of 19 RCTs testing ICIs as monotherapy, the pooled between-groups difference of mean change from baseline to 12 weeks of follow-up was 4.6 (95% CI, 2.8-6.4), and the mean change from baseline to 24 weeks of follow-up was 6.1 (95% CI, 4.2-8.1), significantly favoring ICIs. The pooled difference was 1.4 (95% CI, −0.4 to 3.2) at week 12 and 2.5 (95% CI, −0.8 to 5.9) at week 24 in the group of 8 RCTs testing ICIs combined with chemotherapy and 2.1 (95% CI, −0.8 to 5.0) at week 12 and 2.1 (95% CI, −0.4 to 4.5) at week 24 in the group of 8 RCTs testing other ICI-containing combinations. The time to deterioration was significantly longer in the immunotherapy-containing groups compared with control groups in all 3 groups of RCTs evaluated (hazard ratios of 0.80 [95% CI, 0.70-0.91] for ICIs as monotherapy, 0.89 [95% CI, 0.78-1.00] for ICIs plus chemotherapy, and 0.78 [95% CI, 0.63-0.96] for other ICI-containing combinations).

**Conclusions and Relevance:**

Immune checkpoint inhibitors as monotherapy appear to have a favorable association with patient-reported quality of life and can be combined with other classes of anticancer drugs without worsening this quality of life.

## Key Points

**Question:**

How are immune checkpoint inhibitors in monotherapy or in combination with other anticancer drugs associated with the quality of life of patients with solid tumors?

**Findings:**

In this systematic review and meta-analysis of 34 randomized clinical trials involving 18 709 patients, the pooled between-groups difference of the patient-reported outcomes of mean change from baseline to 12 and 24 weeks of follow-up and time to deterioration favored immunotherapy-containing groups compared with control groups not containing immunotherapy.

**Meaning:**

Immune checkpoint inhibitors have a favorable association with patient quality of life and may be combined with other anticancer drugs without worsening quality of life.

## Teaser

This systematic review and meta-analysis of randomized clinical trials involving patients with solid tumors assesses the association of immune checkpoint inhibitors with patient-reported quality of life.

## Introduction

Immune checkpoint inhibitors (ICIs) have changed the paradigm of treatment of several cancer types. Currently, ICIs are administered as monotherapy or in combination with other immunotherapy drugs or other anticancer agents, such as targeted therapies or chemotherapies.^1,2^ Both the efficacy and toxicity profiles of ICIs meaningfully differ from those of other classes of anticancer treatments.^3,4^ The quality of life (QoL) of patients with metastatic cancer depends on multiple factors, some of which are independent of anticancer treatments, such as socioeconomic background, psychological condition, and concomitance of other chronic diseases, whereas other factors are strictly related to the cancer and its treatment, such as symptoms caused by the tumor that are in turn affected by the efficacy and toxicity of treatments.^5^ Patient-reported outcomes (PROs) are able to capture QoL in a comprehensive way from the patient’s point of view, taking into account all the different aspects that contribute to its definition.^6^ In particular, the time to deterioration (TTD) of PRO score, defined as the time from patient randomization until the first deterioration of PRO score of clinical relevance, is a largely adopted measure to assess treatment effects on patient QoL during the entire trial follow-up, supported by international guidelines.^7^

Although the efficacy of ICIs has been extensively investigated in the past few years, their association with patient QoL, compared with that of other available anticancer treatments, has been less explored. In this report, we detail the results of a systematic review and meta-analysis of PROs assessed in randomized clinical trials (RCTs) testing immunotherapy-based treatments vs anticancer treatments other than immunotherapy for patients with advanced solid tumors.

## Methods

### Search Strategy, Selection Criteria, and Data Extraction

We searched PubMed, MEDLINE, Embase, and Scopus for RCTs testing ICIs and reporting PROs, published from database inception to June 1, 2021. We also reviewed abstracts and presentations from all major conference proceedings, including the American Society of Clinical Oncology and the European Society for Medical Oncology, from January 1, 2010, to June 1, 2021. We followed recommendations of the Preferred Reporting Items for Systematic Reviews and Meta-analyses (PRISMA) reporting guideline and Setting International Standards in Analyzing Patient-Reported Outcomes and Quality of Life Endpoints Data Consortium.^6,8^ This study was exempted from ethics review by the European Institute of Oncology Institutional Review Board because it was a secondary synthesis of deidentified data.

Two investigators (L.P. and F.C.) independently searched the databases. The search terms were *health related quality of life*, *HRQoL*, *patient reported outcomes*, *PROs*, *CTLA-4*, *cytotoxic T-lymphocyte-associated protein 4*, *PD-1*, *programmed death receptor 1*, *immune checkpoint inhibitor*, *ipilimumab*, *tremelimumab*, *nivolumab*, *pembrolizumab*, *durvalumab*, *atezolizumab*, *cemiplimab*, and *spartalizumab*.

We included RCTs that assessed programmed cell death receptor 1, programmed cell death ligand 1 (PD-L1), and cytotoxic T-lymphocyte–associated antigen 4 (CTLA-4) inhibitors as monotherapy or in combination with another ICI and/or other anticancer drugs (ie, targeted therapy or chemotherapy) vs control groups not containing immunotherapy in patients with advanced solid tumors. We excluded single-group phase 1 and 2 trials and RCTs conducted in adjuvant and neoadjuvant settings or in hematologic tumors to avoid excessive heterogeneity. We included trials in which PROs were assessed through the Global Health Status (GHS) scale from the European Organization for Research and Treatment of Cancer (EORTC) Core Quality of Life Questionnaire (QLQ-C30) or the EuroQol Health-Related Quality of Life 5-Dimension, 3-Level (EQ-5D-3L) visual analog scale (VAS).

The GHS scale includes 2 items that explore the patients’ overall health and quality of life. The raw scores are transformed to a linear scale that ranged from 0 to 100. Higher scores on the GHS scale indicate higher levels of health-related quality of life (HRQoL).^9,10,11^ The EQ-5D-3L scale evaluates the patient’s self-rated health state on a 100-point vertical VAS (ie, with 0 indicating worst imaginable health state and 100 indicating best imaginable health state).^12^ We excluded trials reporting PROs only assessed through cancer-specific scales to ensure comparability across trials.^13,14,15,16^

Full-text articles were reviewed independently by 2 authors (L.P. and F.C.). Inconsistencies were discussed by all authors to reach consensus. Reference lists of articles included in the final selection were reviewed to identify additional relevant articles. We included only the most recent and complete report when duplicate publications were identified. We extracted data on the following variables: study’s name, first author and year of publication, study design and blinding, trial phase, underlying malignant neoplasm, number of patients, median follow-up time, treatment groups, line of therapy, PRO scale used, and PRO results.

### Quality Assessment of Trials and PRO Reporting and Data Analysis

To ascertain risk of bias, we assessed the methodologic quality of each trial using the Cochrane Risk of Bias tool, version 5.1.0.^17,18^ The coprimary aims of the meta-analysis were (1) to assess differences between treatment groups in the mean change of PRO score from baseline to 12 and 24 weeks of follow-up assessed through the QLQ-C30 GHS or EQ-5D-3L VAS and (2) to assess differences between treatment groups in the TTD of PRO score, defined as the time from patient randomization until the first deterioration of PRO score that met or exceeded the minimally important difference. As established in previous literature,^9,10,11,12,13,14,15,16^ the minimally important difference indicates a clinically meaningful change of PRO score and was a change of 5 to 10 points for QLQ-C30 GHS and 7 or more points for the Euro-Qol-5 Dimension VAS.

For each end point, trials have been analyzed according to the type of treatment administered in the experimental group: ICIs given as monotherapy, ICIs combined with chemotherapy, or ICIs in association with another ICI and/or with targeted therapies. A sensitivity analysis was performed excluding RCTs whose results were only available as congress abstracts.

### Statistical Analysis

We performed separate meta-analyses of the 3 following end points: (1) the difference in mean change of PRO scores between treatment groups at 12 weeks from baseline, (2) the differences in mean change of PRO scores between treatment groups at 24 weeks from baseline; and (3) the hazard ratio (HRs) for TTD in PROs. Data were retrieved from the original article or reconstructed with validated algorithms.^19,20^ Random-effect models were used to calculate the pooled estimates. Heterogeneity among studies was assessed using the *Q* statistic and *I*^2^ index. A 2-stage meta-analytical approach based on pseudo–individual patient data (IPD)^21^ was used to adjust the pooled difference in mean changes at 12 and 24 weeks for potential baseline imbalances in PRO scores between treatment groups. A 2-sided *P* < .05 was considered statistically significant. All analyses were conducted using SAS software, version 9.4 (SAS Institute Inc) and R software, version 3.6.0 (R Foundation for Statistical Computing). Additional details on statistical analyses are reported in the eMethods in the Supplement.

## Results

Thirty-four RCTs, enrolling a total of 18 709 patients, were included in the analysis (eFigure in the Supplement; Table).^22,23,24,25,26,27,28,29,30,31,32,33,34,35,36,37,38,39,40,41,42,43,44,45,46,47,48,49,50,51,52,53,54,55,56,57^ Twenty-one studies^22,25,30,31,34,40,41,43,44,45,46,47,48,49,50,51,52,53,54,55,56,57^ investigated PROs in the first-line setting, and 13 studies^23,24,26,27,28,29,32,33,35,36,37,38,39,42^ explored PROs in lines beyond first. Nineteen trials^22,23,24,25,26,27,28,29,30,31,32,33,34,35,36,37,38,39,40,41,42^ tested ICIs as monotherapy, 8 trials^43,44,45,46,47,48,49,50^ evaluated the combination of ICIs with chemotherapy, and 8 trials^51,52,53,54,55,56,57^ tested other ICIs-containing combinations.

**Table.** Characteristics of the Studies Included in the Meta-analysis

Study	Trial name	PROs used to assess time to deterioration	PROs used to assess GHS mean change from baseline	Cancer type	Line	Treatment group	No. of patients at risk of deterioration	No. of patients with clinically meaningful deterioration	No. of patients with baseline PRO assessment for GHS mean change analysis	Follow-up duration for analysis of GHS mean change from baseline, wk	End points considered in the meta-analysis		
											Difference in GHS mean change at 12 wk	Difference in GHS mean change at 24 wk	TTD
**ICI monotherapy**														
André et al,^22^ 2020	Keynote 177	QLQ-C30	QLQ-C30	Colon	1	Pembrolizumab	141	30	141	45	Yes	Yes	Yes
						Chemotherapy	131	39	131				
Van Cutsem et al,^23^ 2019	Keynote 061	QLQ-C30	NA	Gastroesophageal	>1	Pembrolizumab	188	NR	NA	NA	No	No	Yes
						Chemotherapy	183	NR	NA				
Harrington et al,^24^ 2020	Keynote 040	QLQ-C30	QLQ-C30	HNSCC	>1	Pembrolizumab	241	117	231	51	Yes	Yes	Yes
						Chemotherapy or targeted therapy	228	113	215				
Long et al,^25^ 2016	CheckMate 066	QLQ-C30	QLQ-C30	Melanoma	1	Nivolumab	147	65	143	43	Yes	Yes	Yes
						Chemotherapy	135	67	135				
Reck et al,^26^ 2018	CheckMate 017	EQ-5D	EQ-5D	NSCLC	>1	Nivolumab	97	48	97	60	Yes	Yes	Yes
						Docetaxel	88	54	89				
Reck et al,^27^ 2018	CheckMate 057	EQ-5D	EQ-5D	NSCLC	>1	Nivolumab	208	121	208	66	Yes	Yes	Yes
						Docetaxel	212	129	212				
Barlesi et al,^28^ 2019	Keynote 010	NA	QLQ-C30	NSCLC	>1	Pembrolizumab	NA	NA	312	12	Yes	No	No
						Chemotherapy	NA	NA	266				
Bordoni et al,^29^ 2018	OAK	QLQ-C30	QLQ-C30	NSCLC	>1	Atezolizumab	421	133	410	39	Yes	Yes	Yes
						Chemotherapy	400	102	387				
Hui et al,^30^ 2019	PACIFIC	QLQ-C30	QLQ-C30	NSCLC	1	Durvalumab	470	274	474	48	Yes	Yes	Yes
						Placebo	232	129	232				
Brahmer et al,^31^ 2017	Keynote 024	NA	QLQ-C30	NSCLC	1	Pembrolizumab	NA	NA	145	33	Yes	Yes	No
						Chemotherapy	NA	NA	138				
Vaughn et al,^32^ 2018	Keynote 045	QLQ-C30	QLQ-C30	Urothelial	>1	Pembrolizumab	260	154	260	51	Yes	Yes	Yes
						Chemotherapy	242	148	242				
Powles et al,^33^ 2017	IMvigor 211	QLQ-C30	NA	Urothelial	>1	Atezolizumab	440	157	NA	No	No	No	Yes
						Chemotherapy	422	125	NA				
Van Cutsem et al,^34^ 2019	Keynote 062	QLQ-C30	NA	Gastroesophageal	1	Pembrolizumab	252	NR	NA	No	No	No	Yes
						Chemotherapy	243	NR	NA				
Harrington et al,^35^ 2017	CheckMate 141	QLQ-C30	NA	HNSCC	>1	Nivolumab	240	49	NA	NA	NA	NA	No
						Chemotherapy	121	34	NA				
Ferris et al,^36^ 2016	CheckMate 141	NA	QLQ-C30	HNSCC	>1	Nivolumab	NA	NA	191	21	Yes	NA	No
						Chemotherapy	NA	NA	91				
Ryoo et al,^37^ 2020	Keynote 240	NA	QLQ-C30	HCC	>1	Pembrolizumab and best supportive care	NA	NA	271	45	Yes	Yes	No
						Placebo and best supportive care	NA	NA	127				
Larkin et al,^38^ 2018	CheckMate 037	NA	QLQ-C30	Melanoma	>1	Nivolumab	NA	NA	272	66	Yes	Yes	No
						Chemotherapy	NA	NA	133				
Schadendorf et al,^39^ 2016	Keynote 002	NA	QLQ-C30	Melanoma	>1	Pembrolizumab, 2 mg/kg	NA	NA	169	36	Yes	Yes	No
						Pembrolizumab, 10 mg/kg	NA	NA	168				
						Chemotherapy	NA	NA	155				
Sezer et al,^40,41^ 2021	EMPOWER-Lung 1	NA	QLQ-C30	NSCLC	1	Cemiplimab	NA	NA	331	78	Yes	Yes	No
						Chemotherapy	NA	NA	309				
Cella et al,^42^ 2016	CheckMate 025	NA	EQ-5D	RCC	>1	Nivolumab	NA	NA	361	104	Yes	Yes	No
						Targeted therapy	NA	NA	344				
**ICI and chemotherapy**														
Adams et al,^43^ 2020	IMpassion 130	QLQ-C30	QLQ-C30	Breast	1	Atezolizumab and chemotherapy	403	212	403	136	Yes	Yes	Yes
						Chemotherapy	397	200	397				
Mazieres et al,^44^ 2020	Keynote 407	NA	QLQ-C30	NSCLC	1	Pembrolizumab and chemotherapy	NA	NA	254	36	Yes	Yes	No
						Chemotherapy	NA	NA	264				
Garassino et al,^45^ 2020	Keynote 189	NA	QLQ-C30	NSCLC	1	Pembrolizumab and chemotherapy	NA	NA	359	30	Yes	Yes	No
						Chemotherapy	NA	NA	180				
Kim et al,^46^ 2020	Keynote 604	QLQ-C30	QLQ-C30	SCLC	1	Pembrolizumab and chemotherapy	221	44	208	18	Yes	No	Yes
						Chemotherapy	218	54	204				
Bamias et al,^47^ 2020	IMvigor 130	QLQ-C30	QLQ-C30	Urothelial	1	Atezolizumab and chemotherapy	451	140	362	96	Yes	Yes	Yes
						Chemotherapy	400	136	327				
Goldman et al,^48^ 2020	CASPIAN	QLQ-C30	QLQ-C30	SCLC	1	Durvalumab and chemotherapy	268	133	245	45	Yes	Yes	Yes
						Chemotherapy	269	109	245				
Reck et al,^49^ 2020	IMpower 150	NA	QLQ-C30	NSCLC	1	Atezolizumab and chemotherapy	NA	NA	371	36	Yes	Yes	No
						Targeted therapy and chemotherapy	NA	NA	360				
Mansfield et al,^50^ 2020	IMpower 133	NA	QLQ-C30	SCLC	1	Atezolizumab and chemotherapy	NA	NA	179	54	Yes	Yes	No
						Chemotherapy	NA	NA	175				
**Other ICI-containing combinations**														
Reck et al,^51^ 2019	CheckMate 227	EQ-5D	EQ-5D	NSCLC	1	Nivolumab and ipilimumab	139	42	113	84	Yes	Yes	Yes
						Chemotherapy	160	69	141				
Cella et al,^52^ 2019	CheckMate 214	EQ-5D	EQ-5D	RCC	1	Ipilimumab and nivolumab	425	NR	415	103	Yes	Yes	Yes
						Targeted therapy	422	NR	403				
Sherpereel et al,^53^ 2020	CheckMate 743	EQ-5D	NA	Mesothelioma	1	Nivolumab and ipilimumab	303	NR	NA	No	No	No	Yes
						Chemotherapy	302	NR	NA				
Reck et al,^54^ 2020	CheckMate 9LA	EQ-5D	EQ-5D	NSCLC	1	Ipilimumab, nivolumab, and chemotherapy	361	NR	330	78	Yes	Yes	Yes
						Chemotherapy	358	NR	321				
Reck et al,^49^ 2020	IMpower 150	NA	QLQ-C30	NSCLC	1	Atezolizumab, targeted therapy, and chemotherapy	NA	NA	356	36	Yes	Yes	No
						Targeted therapy and chemotherapy	NA	NA	360				
Finn et al,^55^ 2020	IMbrave 150	QLQ-C30	NA	HCC	1	Atezolizumab and targeted therapy	336	132	NA	No	No	No	Yes
						Targeted therapy	165	68	NA				
Lewis et al,^56^ 2020	IMspire 150	QLQ-C30	NA	Melanoma	1	Atezolizumab and targeted therapy	256	91	NA	No	No	No	Yes
						Targeted therapy	258	77	NA				
Bedke,^57^ 2020	Keynote 426	EQ-5D	QLQ-C30	RCC	1	Pembrolizumab and targeted therapy	428	NR	394	30	Yes	Yes	Yes
						Targeted therapy	423	NR	410				

Abbreviations: EQ-5D, EuroQol Health-Related Quality of Life 5-Dimension; GHS, Global Health Status; HCC, hepatocellular carcinoma; HNSCC, head and neck squamous cell carcinoma; ICI, immune checkpoint inhibitor; NA, not applicable; NR, not reported; NSCLC, non–small cell lung cancer; PRO, patient-reported outcome; QLQ-C30, European Organization for Research and Treatment of Cancer (EORTC) Core Quality of Life Questionnaire; RCC, renal carcinoma; SCLC, small cell lung cancer; TTD, time to deterioration.

The experimental group was an anti-PD1 or anti–PD-L1 drug given as monotherapy in 19 trials,^22,23,24,25,26,27,28,29,30,31,32,33,34,35,36,37,38,39,40,41,42^ an anti-PD1 or anti–PD-L1 drug combined with chemotherapy in 8 trials,^43,44,45,46,47,48,49,50^ an anti-PD1 or anti–PD-L1 drug combined with targeted therapy in 3 trials,^55,56,57^ and the combination of an anti-PD1 with an anti-CTLA4 drug in 3 trials.^51,52,53^ Combination immunotherapy (ie, anti-PD1 and anti-CTLA4 drug) plus chemotherapy and an anti–PD-L1 combined with both chemotherapy and targeted therapy was the experimental group in 1 trial each.^49,54^ Twelve trials^26,27,28,29,30,31,40,41,44,45,49,51,54^ were conducted in patients with non–small cell lung cancer; 4 trials^25,38,39,56^in patients with melanoma; 3 trials each in patients with small cell lung cancer,^46,48,50^ renal carcinoma,^42,52,57^ and urothelial carcinoma^32,33,47^; and 2 trials each in patients with head and neck squamous cell carcinomas,^24,35,36^ hepatocellular carcinoma,^37,55^ and gastroesophageal cancer^23,34^; and 1 trial each enrolled patients with colon cancer,^22^ breast cancer,^43^ and mesothelioma.^53^ Median follow-up of trials was 46.5 weeks (ranging from 12 to 136 weeks).

eTable 1 in the Supplement reports the quality assessment of trials according to the Cochrane Risk of Bias tool. Overall, the quality of trials was high because the risks of selection, attrition, reporting, and other forms of bias for all the RCTs included in the analysis were low. The only potential biases that affected the trials were performance and detection bias because only 12 of 34 RCTs^22,24,31,32,33,38,49,51,53,54,55,57^ had a double-blinding design. The quality assessment of PRO reporting for each trial is presented in eTable 2 in the Supplement. The median score was 4 (ranging from 2 to 5), and only 3 RCTs^33,38,55^ obtained a low score (ie, <3).

In the group of 19 trials testing ICIs as monotherapy, the mean change of PRO score from baseline to 12 and 24 weeks of follow-up was reported in 16 trials^22,24,25,26,27,28,29,30,31,32,36,37,38,39,40,41,42^ and 14 trials,^22,24,25,26,27,29,30,31,32,37,38,39,40,41,42^ respectively, and was assessed by the EORTC QLQ-C30 GHS in 13 trials^22,24,25,28,29,30,31,32,36,37,38,39,40,41^ and by the EQ-5D-3L VAS in 3 trials.^26,27,42^ One trial^39^ had 2 groups that contained immunotherapy evaluated separately. All such RCTs were included in the analysis, for a total number of 7390 individual PRO assessments recorded at baseline and at 12 weeks of follow-up (16 RCTs,^22,24,25,26,27,28,29,30,31,32,36,37,38,39,40,41,42^ 17 pairwise comparisons between groups) and 6530 at 24 weeks (14 RCTs,^22,24,25,26,27,29,30,31,32,37,38,39,40,41,42^ 15 pairwise comparisons between groups).

**Figure 1.**
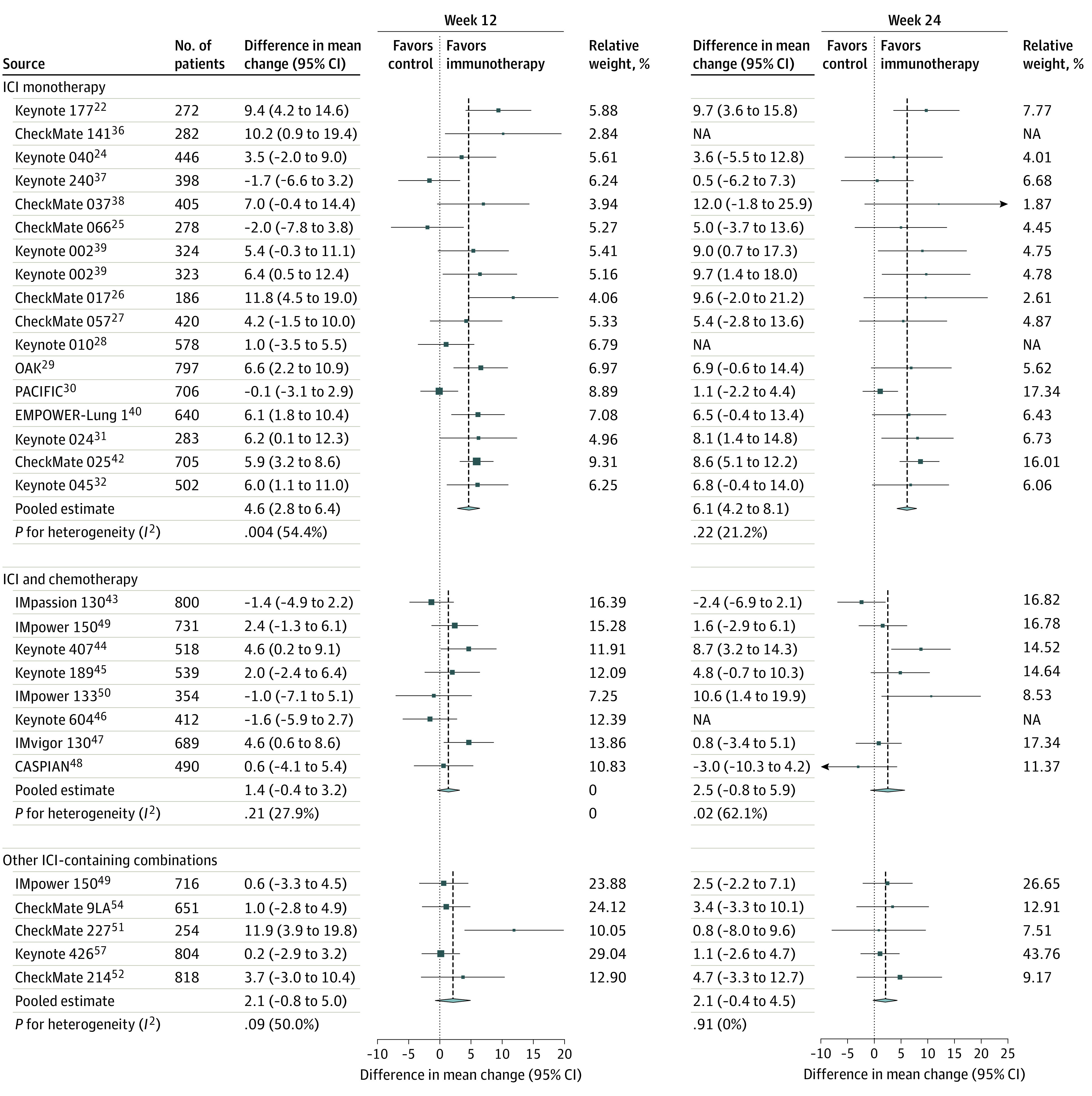
Between-Groups Differences in Mean Change of Patient-Reported Outcomes (PROs) From Baseline to 12 Weeks and to 24 Weeks According to Experimental Treatment Groups The between-groups differences in mean change of PROs assessed from baseline to 12 weeks or 24 weeks of follow-up are shown for patients assigned to intervention treatment (ie, immunotherapy-containing groups) compared with those assigned to control treatment (ie, groups not containing immunotherapy). Studies are grouped according to the experimental group type of treatment (ie, immune checkpoint inhibitor [ICI] monotherapy, ICI and chemotherapy, other ICI-containing combinations). Squares indicate study-specific mean change difference of PROs between treatment groups. Values higher than 0 indicate that the intervention was better than the control. Square size is proportional to the precision of the estimate (ie, the inverse of the variance). Horizontal lines indicate the 95% CIs. Diamonds indicate the meta-analytic pooled mean change differences of PROs between treatment groups, according to experimental treatment groups, calculated at 12 and 24 weeks of follow-up, with their corresponding 95% CIs. The dashed vertical lines indicate the pooled differences in mean change, and the dotted vertical line indicates a mean change difference of 0, which is the null-hypothesis value (ie, no difference between treatment groups). NA indicates not applicable.

**Figure 2.**
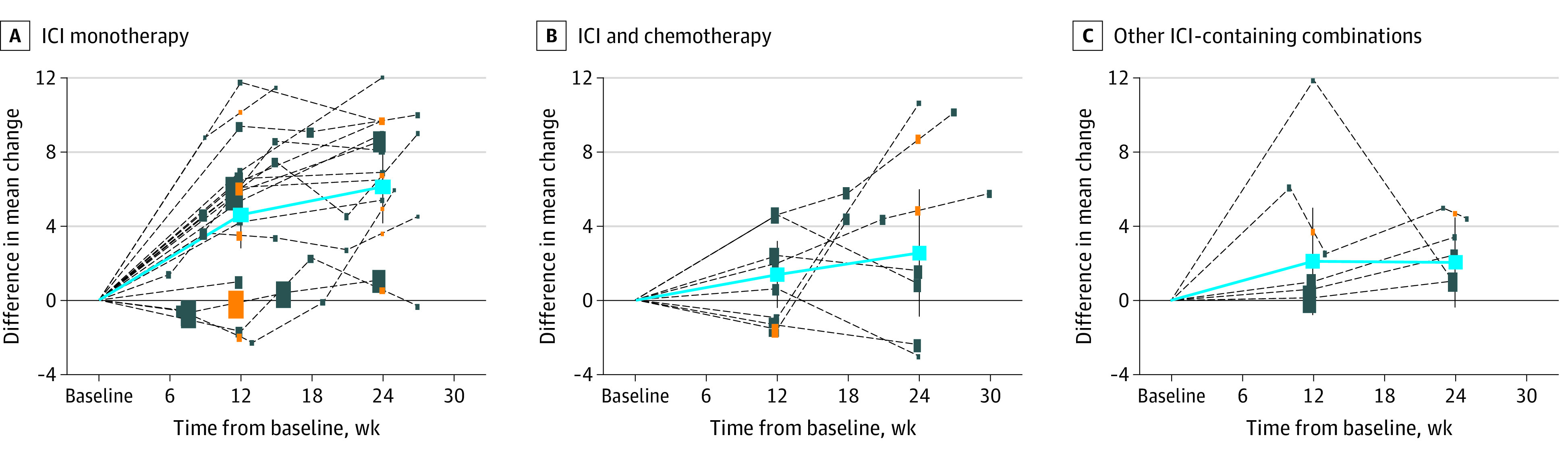
Trajectories Over Time of Between-Groups Differences in Mean Change of Patient-Reported Outcomes (PROs) Assessed in Each Trial and Pooled Estimates According to Experimental Treatment Groups The difference in mean change of PROs are shown for each treatment comparison (dark blue dashed lines and boxes) and the meta-analytic pooled estimates (solid blue line and boxes) according to experimental treatment groups with corresponding 95% CIs (ie, immune checkpoint inhibitor [ICI] monotherapy, ICI and chemotherapy, and other ICI-containing combinations). Each dashed line represents a single treatment comparison, and the size of each rectangle reflects the precision of each effect. For trials in which comparisons at 12 and 24 weeks of follow-up were not reported or derivable (orange boxes), these values were estimated using the information at the previous and subsequent available time points. Values below the solid horizontal line favor the control, and values above the line favor immunotherapy.

The between-groups difference of mean change in PRO score from baseline to 12 weeks and 24 weeks of follow-up favored the immunotherapy-containing group in 14 of 17 pairwise comparisons at 12 weeks and in 15 of 15 pairwise comparisons at 24 weeks (Figure 1 and Figure 2). The pooled between-groups difference of mean change in PRO score from baseline was 4.6 (95% CI, 2.8-6.4) at week 12 and 6.1 (95% CI, 4.2-8.1) at week 24, favoring immunotherapy-containing groups (Figure 1 and Figure 2). There was significant heterogeneity among single-study estimates at 12 weeks (*I*^2^ = 54.4%, *P* = .004), which became small and not significant at 24 weeks of follow-up (*I*^2^ = 21.2%, *P* = .22) (Figure 1 and Figure 2).

In the group of 8 trials testing ICIs in combination with chemotherapy, the mean change in PRO score from baseline to 12 and 24 weeks of follow-up was reported in all 8 trials^43,44,45,46,47,48,49,50^ at 12 weeks and 7 trials^43,44,45,47,48,49,50^ at 24 weeks and was assessed by the EORTC QLQ-C30 GHS in all the trials.^43,44,45,46,47,48,49,50^ All such RCTs were included in the analysis for a total number of 4533 individual PRO assessments recorded at baseline and at 12 weeks of follow-up (8 RCTs,^43,44,45,46,47,48,49,50^ 8 pairwise comparisons between groups) and 4121 at 24 weeks (7 RCTs,^43,44,45,47,48,49,50^ 7 pairwise comparisons between groups).

The between-groups difference of mean change of PRO score from baseline to 12 weeks and 24 weeks of follow-up favored the immunotherapy-containing group in 5 of 8 pairwise comparisons at 12 weeks and in 5 of 7 pairwise comparisons at 24 weeks (Figure 1 and Figure 2). The pooled between-groups difference of mean change in PRO scores from baseline was 1.4 (95% CI, −0.4 to 3.2) at week 12 and 2.5 (95% CI, −0.8 to 5.9) at week 24, favoring immunotherapy-containing groups (Figure 1 and Figure 2). Small and not significant heterogeneity was found among single-study estimates at 12 weeks (*I^2^* = 27.9%, *P* = .21), which became significant at 24 weeks of follow-up (*I*^2^ = 62.1%, *P* = .02) (Figure 1 and Figure 2).

In the group of 8 trials^49,51,52,53,54,55,56,57^ testing other ICI-containing combinations, the mean change in PRO score from baseline to 12 and 24 weeks of follow-up was reported for both time points in 5 trials^49,51,52,54,57^ and was assessed by the EORTC QLQ-C30 GHS in 2 trials^49,57^ and by the EQ-5D-3L VAS in 3 trials.^51,52,54,57^

All such RCTs were included in the analysis, for a total of 3243 individual PRO assessments recorded at baseline and at 12 weeks of follow-up (5 RCTs, 5 pairwise comparisons between groups) and 3243 at 24 weeks (5 RCTs, 5 pairwise comparisons between groups). The between-groups difference of mean change in PRO score from baseline to 12 and 24 weeks of follow-up favored the immunotherapy-containing group in 4 of 5 pairwise comparisons at 12 weeks and in 5 of 5 pairwise comparisons at 24 weeks (Figure 1 and Figure 2). The pooled between-groups difference of mean change in PRO score from baseline was 2.1 (95% CI, −0.8 to 5.0) at week 12 and 2.1 (95% CI, −0.4 to 4.5) at week 24, favoring immunotherapy-containing groups (Figure 1 and Figure 2). There was no significant heterogeneity among single-study estimates at 12 weeks (*I*^2^ = 50.0%; *P* = .09); this finding became null at 24 weeks of follow-up (*I*^2^ = 0.0%, *P* = .91) (Figure 1 and Figure 2).

To adjust the overall pooled treatment effect for potential imbalance of PRO baseline scores between treatments, a 2-stage meta-analysis based on pseudo-IPD was conducted. In the group of trials testing ICIs as monotherapy, the adjusted pooled effects were 5.2 (95% CI, 3.5-6.8) at 12 weeks and 7.l (95% CI, 5.3-8.9) at 24 weeks. In the group of trials testing ICIs in combination with chemotherapy, the adjusted pooled effects were 1.9 (95% CI, 0.1-3.6) at 12 weeks and 3.2 (95% CI, −0.2 to 6.5) at 24 weeks. Finally, in the group of trials testing other ICI-containing combinations, the adjusted pooled effects were 3.5 (95% CI, 0.2-6.7) at week 12 and 2.9 (95% CI, 0.8-5.1) at week 24.

The TTD of PROs was reported in 23 of 34 RCTs (12 RCTs testing ICIs as monotherapy,^22,23,24,25,26,27,29,30,32,33,34,35^ 4 trials testing ICIs combined with chemotherapy,^43,46,47,48^ and 7 trials testing other ICI-containing combinations^51,52,53,54,55,56,57^). The TTD was assessed through EORTC QLQ-C30 GHS in 16 trials^22,23,24,25,29,30,32,33,34,35,43,46,47,48,55,56^ and EQ-5D-3L^26,27,51,52,53,54,57^ VAS in 7 trials.

**Figure 3.**
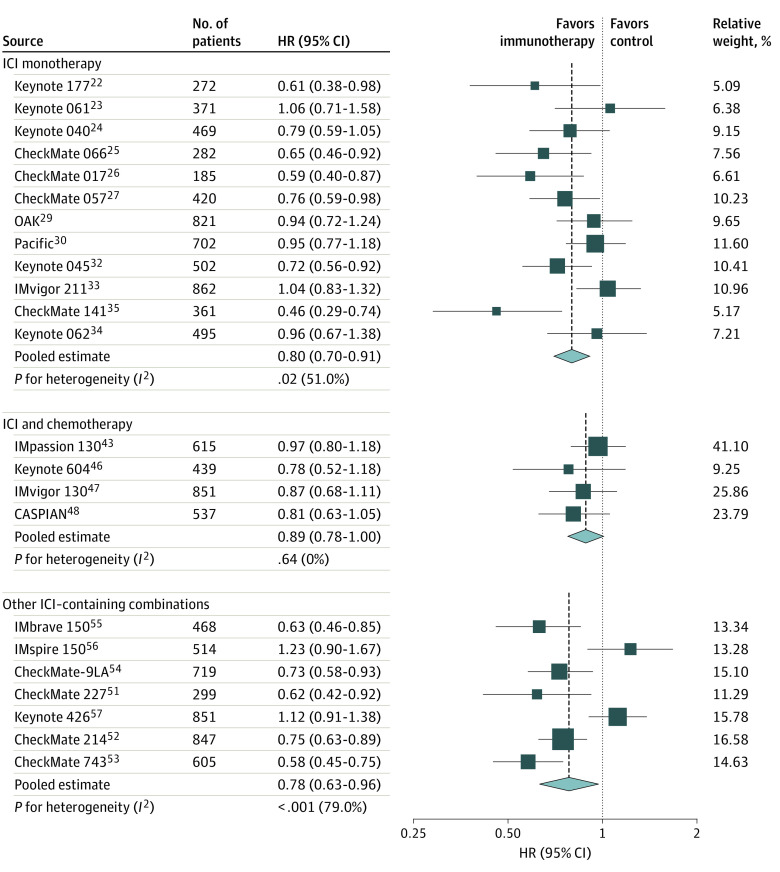
Hazard Ratios for Time to Deterioration According to Experimental Treatment Groups The hazard ratios (HRs) of time to deterioration for patients assigned to intervention treatment (ie, immunotherapy-containing groups) compared with those assigned to control treatment (ie, groups not containing immunotherapy) are shown. Studies are grouped according to the experimental group type of treatment (ie, immune checkpoint inhibitor [ICI] monotherapy, ICI and chemotherapy, and other ICI-containing combinations). Squares indicate study specific HRs. Values less than 1 indicate that intervention was better than the control. Size of the square is proportional to the precision of the estimate (ie, the inverse of the variance). Horizontal lines indicate the 95% CIs. Diamonds indicate the meta-analytic pooled HRs, with their corresponding 95% CIs. The dashed vertical lines indicate the pooled HRs, and the dotted vertical line indicates an HR of 1, which is the null-hypothesis value (ie, no difference in time to deterioration between treatment groups).

In the group of trials testing ICIs as monotherapy, the TTD was longer in the immunotherapy-containing groups compared with control groups in 10 of 12 RCTs^22,24,25,26,27,29,30,32,34,35^ (pooled TTD HR, 0.80; 95% CI, 0.70-0.91) (Figure 3). Significant heterogeneity was found among single-study estimates of TTD (*I*^2^ = 51.0%, *P* = .02). In the group of trials testing ICIs in combination with chemotherapy, the TTD was longer in the immunotherapy-containing groups compared with control groups in all trials (pooled TTD HR, 0.89; 95% CI, 0.78-1.00) (Figure 3). No heterogeneity was found among single-study estimates of TTD (*I*^2^ = 0.0%, *P* = .64). In the group of trials testing other ICI-containing combinations, the TTD was longer in the immunotherapy-containing groups compared with control groups in 5 of 7 RCTs^51,52,53,54,55^ (pooled TTD HR, 0.78; 95% CI, 0.63-0.96) (Figure 3). Significant heterogeneity was found among single-study estimates of TTD (*I*^2^ = 79.0%, *P* < .001).

Finally, a sensitivity analysis was performed excluding RCTs whose results were only available as congress abstracts. Results did not materially change compared with those of the main analyses for both the mean change in PRO score at 12 and 24 weeks and the TTD (eTable 3 in the Supplement).

## Discussion

We assessed the association of ICIs with the quality of life of more than 18 000 patients with solid tumors treated in 34 RCTs. Notably, even though few studies^58,59^ have been conducted in this area, to our knowledge, this meta-analysis is the largest and includes only RCTs. Furthermore, we provided evidence on the association of recent ICI-containing treatments on PROs, especially of the combination of ICIs and chemotherapy, which is becoming a standard therapeutic approach for a large number of solid tumors.

Our results clearly show that differences in PROs over time favor immunotherapy in trials testing ICI monotherapy. However, in trials testing ICI-containing combinations, the degree of PRO improvement in favor of immunotherapy at 12 or 24 weeks was limited and under the clinically relevant cutoff. Although this result does not allow for the conclusion of better HRQoL in patients treated with an ICI combination, it supports the conclusion that none of the multidrug combinations worsened patient quality of life compared with control groups. This finding is noteworthy considering that in some RCTs, patients received up to 3 different classes of drugs.

A significantly longer preservation of quality of life for patients treated with immunotherapy-containing treatments, including multidrug combinations, is further supported by the results of TTD analysis, which captures HRQoL during the entire trial follow-up and not only at specific time points. This finding could be partially explained by the longer disease control achieved in many trials by patients receiving ICIs compared with the control group as well as by the characteristic toxicity profile of this new class of drugs.

Indeed, as a consequence of the meaningful immunotherapy efficacy, a large number of patients randomized to an ICI-containing group did not withdraw and provided PRO assessments for a long period. The spectrum of adverse events of ICIs is different from that of all other systemic therapies, and many patients develop no or mild adverse events that do not substantially affect quality of life. This difference could explain the results of the CheckMate 9LA trial,^54^ in which patients with advanced non–small cell lung cancer treated with the combination of chemotherapy plus nivolumab and ipilimumab experienced a significantly longer TTD compared with those receiving only chemotherapy. Similarly, in the IMbrave150 trial,^55^ patients with advanced hepatocarcinoma treated with the combination of atezolizumab plus bevacizumab had a significantly longer TTD compared with the control group.

Some exceptions have been reported. For example, the IMspire150 trial^56^ showed an increased risk of quality-of-life deterioration for patients with melanoma who received ICIs in combination with anti-*BRAF* and anti-MEK targeted therapy because of the high risk of adverse events reported for this specific combination of drugs.

An important observation that emerged from our systematic review is that none of the considered RCTs included HRQoL as the primary end point, and often PROs were reported only in secondary and delayed reports. This observation highlights the underestimation of the importance of HRQoL in the field of anticancer immunotherapy.

Several measures should be enacted to improve HRQoL assessment for immunotherapy. The assessment of HRQoL should be included within the primary objectives of RCTs testing immunotherapy. Furthermore, to achieve an unbiased assessment of the risk-benefit ratio of new therapeutic approaches, patient perception of how therapies impact their quality of life, elicited through PROs, should not be separated from the main analysis of trial results. In this regard, combined end points that jointly evaluate efficacy, toxicity, and HRQoL, such as Q-TWiST (Quality-Adjusted Time Without Symptoms or Toxicity), should be more broadly considered.^60^ Moreover, in most cases, the HRQoL evaluations in RCTs stopped at 24 weeks of follow-up, leaving an important gap in the knowledge of HRQoL of patients surviving in the long term. Because the percentage of long-term survivors has been significantly increased by ICIs, a substantial time extension of HRQoL collection during the follow-up should be planned by trials testing ICIs.^61^ Finally, a paramount limitation of instruments currently in use for assessing PROs is that these instruments have not been specifically developed and validated to evaluate HRQoL in trials testing immunotherapies. Consequently, they may not be able to fully capture peculiar features of tolerability of such new therapies.^9,10,11,12,13,14,15,16^ Scientific societies focused on HRQoL should thus urgently develop, validate, and spread new instruments dedicated for immunotherapy trials.

### Limitations

This work has several limitations. We analyzed published data rather than IPD. However, this weakness was substantially attenuated by the use of reconstructed IPD.^21^ Furthermore, although we found no heterogeneity among single-study estimates in many analyses, there was heterogeneity in others. Such heterogeneity could be related to the different tumor histotypes in the patients enrolled in the RCTs analyzed. Indeed, some dimensions of quality of life may be specifically affected by tumor histotypes. For some cancer histotypes, only a few RCTs were available, which precluded the possibility of performing subgroup analyses. We addressed this issue by using random-effects models that took into account heterogeneity. However, potential differences among patients with different tumor histotypes should be more granularly investigated by future studies. Additionally, because results from only a few RCTs testing ICIs in the neoadjuvant or adjuvant setting have been reported to date, we decided not to include them in our analysis to avoid additional heterogeneity. Thus, the conclusions of our work should be limited to patients treated with ICIs in the advanced disease setting.

## Conclusions

The results of this meta-analysis demonstrate a favorable association of ICIs with patient quality of life compared with control groups that did not contain immunotherapy across a large spectrum of solid tumors. The benefit was particularly evident when ICIs were administered as monotherapy. In addition, this meta-analysis found that ICIs can be combined with several other classes of anticancer drugs, particularly chemotherapy, without worsening patient quality of life, which is a noteworthy finding considering that such combinations will be increasingly used in many solid tumors. Future research should incorporate PROs as a primary end point of RCTs testing immunotherapy to concretely develop a patient-centered model of care.

## Notes

Supplement. **eMethods.** Statistical Analysis **eFigure.** Prisma Flowchart **eTable 1.** Assessment of Risk of Bias in RCTs Included in the Analysis **eTable 2.** Assessment of Quality of PROs Reporting in RCTs Included in the Analysis **eTable 3.** Sensitivity Analysis of TTD and GHS Mean Change Excluding RCTs Only Available as Congress Abstracts
eReferences
