# Error in Author Affiliation

**DOI:** 10.1001/jamanetworkopen.2022.50037

**Published:** 2022-12-15

In the Original Investigation titled “Association of Anticancer Immune Checkpoint Inhibitors With Patient-Reported Outcomes Assessed in Randomized Clinical Trials: A Systematic Review and Meta-analysis,”^1^ published August 16, 2022, author Fabio Conforti’s affiliations incorrectly included the Oncology Unit, Humanitas, Gavalleni, Bergamo, Italy. Dr Conforti’s correct affiliation is the Division of Melanoma, Sarcomas, and Rare Tumors, European Institute of Oncology, Milan, Italy. This article has been corrected.^1^
