# Supplementary material for: Association of Anticancer Immune Checkpoint Inhibitors With Patient-Reported Outcomes Assessed in Randomized Clinical Trials: A Systematic Review and Meta-analysis
Source: JAMA Netw Open. 2022 Aug 16;5(8):e2226252. doi: 10.1001/jamanetworkopen.2022.26252 (PMC9382448; doi:10.1001/jamanetworkopen.2022.26252)
Supplement: Supplement. — eMethods. Statistical Analysis eFigure. Prisma Flowchart eTable 1. Assessment of Risk of Bias in RCTs Included in the Analysis eTable 2. Assessment of Quality of PROs Reporting in RCTs Included in the Analysis eTable 3. Sensitivity Analysis of TTD and GHS Mean Change Excluding RCTs Only Available as Congress Abstracts eReferences [file jamanetwopen-e2226252-s001.pdf]

Supplementary Online Content

Pala L, Sala I, Oriecuia C, et al. Association of anticancer immune checkpoint inhibitors with patient-reported outcomes assessed in randomized clinical trials: a systematic review and meta-analysis. *JAMA Netw Open*. 2022;5(8):e2226252. doi:10.1001/jamanetworkopen.2022.26252

eMethods. Statistical Analysis

eFigure. Prisma Flowchart

eTable 1. Assessment of Risk of Bias in RCTs Included in the Analysis

eTable 2. Assessment of Quality of PROs Reporting in RCTs Included in the Analysis

eTable 3. Sensitivity Analysis of TTD and GHS Mean Change Excluding RCTs Only Available as Congress Abstracts

eReferences

This supplementary material has been provided by the authors to give readers additional information about their work.

© 2022 Pala L et al. *JAMA Network Open.*

eMethods. Statistical analysis

Meta-analysis of difference in mean changes

We performed two separate meta-analyses, considering as effect size of interest the difference between the intervention group *I* and control group *C* of the mean change of PROs score from baseline to 12 weeks, and the difference from baseline to 24 weeks. We extracted, for each study, the mean change at week *w* in the intervention group $d_{I(w)}$ and in the control group $d_{C(w)}$, along with the standard deviations of the individual changes $SD(d_{I(w)})$ and $SD(d_{C(w)})$. The standard error of the mean change in the intervention group was calculated as $SE(d_{I(w)})=SD(d_{I(w)})/\sqrt{n_{I(w)}}$, with $n_{I(w)}$ the number of respondents in the intervention arm at the week *w* of interest; $SE(d_{C(w)})$ was similarly derived. When in the intervention group or in the control group n_w was not reported, we imputed it multiplying the number of patients n_0 evaluated at baseline (usually reported in the paper) by the average proportion of respondents at time *w* calculated from all the other studies reporting n_w.

For each study, we estimated the effect size as $\delta_w=(d_{I(w)}-d_{C(w)})$, and its standard error as $SE(\delta_w)=\sqrt{SE(d_{I(w)})^2+SE(d_{C(w)})^2}$. For studies reporting only the mean μ_w and the standard deviation SD_w of the PRO scores for each arm separately, we calculated the mean change at week *w* as $d_w=\mu_w-\mu_0$. The standard deviation of the individual changes was calculated taking into account the correlation between the paired measures, as $SD(d_w)=\sqrt{SD_0^2+SD_w^2-2\rho SD_0 SD_w}$. The correlation coefficient ρ was imputed as the average coefficient derived from the studies reporting the *SD* of the scores both at baseline and at week *w*, and the SD of the mean difference between week *w* and baseline. When the arm-specific mean changes or the absolute values were reported only in line plots showing the time-point estimates and their 95% confidence intervals (CIs), we digitized the plots and extracted the relevant information, using the software WebPlotDigitizer. (20) For studies in which mean PROs information were presented only for weeks from baseline different than the week of interest (i.e. 12th or the 24th),

© 2022 Pala L et al. *JAMA Network Open.*

and in which the values relative to a previous and a subsequent week, with respect to the week of interest, were both presented, $d_{I(w)}$ and $d_{C(w)}$, and the corresponding $SE(d_{I(w)})$ and $SE(d_{C(w)})$ were imputated. The imputed value was calculated as the weighted mean of the two values relative to the previous and the subsequent weeks, with weights inversely proportional to the distance from the week of interest being estimated and the two available ones.

To estimate the pooled difference in mean changes at 12 and 24 weeks, we used a random-effects model, weighting each study estimate by the inverse of its variance. A pooled difference in mean change greater than 0 indicated a greater benefit in PROs score for the immunotherapy-containing arm. We also calculated the I^2 statistics, which express the percentage of the total observed variability due to heterogeneity between studies' results, and the Q statistic to test the null hypothesis of homogeneity between studies.

To adjust the overall pooled difference in mean changes at 12 and 24 weeks for potential baseline imbalances in PRO scores between treatment and control groups, we used a two-stage meta-analytical approach based on pseudo individual patient data (IPD), as described by Papadimitropoulou et al. (21) This approach proceeded as follows: for each included study, we first constructed the pseudo IPD using the means and the standard deviations of PRO scores reported at baseline and at the follow-up week of interest. Then, in the first meta-analytical stage, we fitted, for each of the N included studies, a linear model with the follow-up score as dependent variable and the treatment and the baseline score as independent variables. This yields N treatment effects δ_i with standard error SE_i, adjusted for baseline imbalance. At the second stage, a random-effects meta-analysis was run on the estimated study specific δ_i to estimate the pooled adjusted treatment effect.

© 2022 Pala L et al. *JAMA Network Open.*

Meta-analysis of the time to deterioration hazard ratio.

The hazard ratios (HRs) for TTD in the treatment arm compared with those in the control arm, along with their 95% CIs, were extracted from each single study. For studies reporting only the Kaplan-Meier (KM) curves for TTD we use the algorithm proposed by Guyot et al. (3) to reconstruct the individual patient time data from published curves. The software WebPlotDigitizer (1) was used to digitize the KM curves. A Cox proportional hazard model was fitted to the reconstructed data to estimate the HR for TTD and the corresponding 95% confidence interval.

HRs and 95% CIs were translated into log-hazard ratios (logHRs), and the corresponding variances were calculated. Overall and subgroup-specific pooled hazard ratios of TTD were calculated using random-effects models. The weight associated to each study was given by the inverse of the study logHR variance. A pooled HR lower than 1 indicates a longer TTD for the immunotherapy-containing arm. I^2 and Q statistics were also calculated.

Finally, a sensitivity analysis was performed excluding RCTs only available as congress abstracts. Pooled HR-TTD and pooled difference in mean change at 12 and 24 weeks were provided.

© 2022 Pala L et al. *JAMA Network Open.*

eFigure 1. PRISMA flow chart

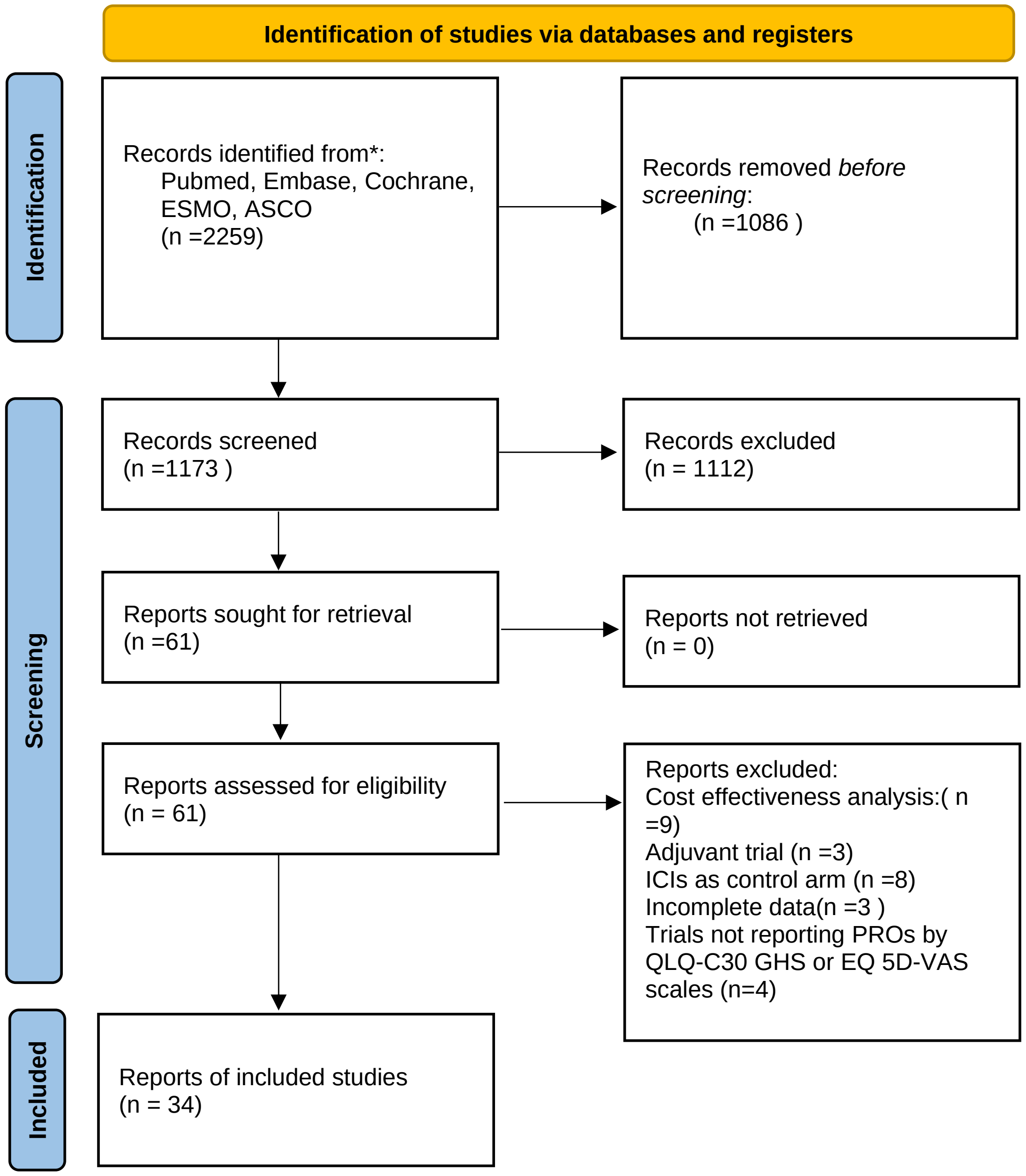

© 2022 Pala L et al. *JAMA Network Open.*

eTable 1. Assessment of risk of bias in RCTs included in the analysis.

Study	Random sequence generation (selection bias)	Allocation concealment (selection bias)	Blinding of partecipants and personnel (performance bias)	Blinding of outcome assessment (detection bias)	Incomplete outcome of data (attrition bias)	Selective reporting (reporting bias)	Other bias
Andre T, 2020 (4)	Low	Low	High	Low	Low	Low	Low
Van Cutsem E, 2019 (5)	Low	Low	Low	Low	Low	Low	Low
Harrington KJ, 2020 (6)	Low	Low	High	Low	Low	Low	Low
Long GV, 2016 (7)	Low	Low	Low	Unclear	Low	Low	Low
Reck M, 2018 (017) (8)	Low	Low	High	Unclear	Low	Low	Low
Reck M, 2018 (057) (9)	Low	Low	High	Unclear	Low	Low	Low
Barlesi F, 2018 (10)	Low	Low	High	Unclear	Low	Low	Low
Bordoni R, 2018 (11)	Low	Low	High	Unclear	Low	Low	Low
Hui R, 2019 (12)	Low	Low	Low	Low	Low	Low	Low
Brahmer JR, 2017 (13)	Low	Low	High	Low	Low	Low	Low
Vaughn DJ, 2018 (14)	Low	Low	High	Low	Low	Low	Low
Powles T, 2017 (15)	Low	Low	High	High	Low	Low	Low
Van Cutsem E, 2019; (062) (16)	Low	Low	Low	Low	Low	Low	Low
Harrington KJ, 2017; Ferris R, 2016 (17,18)	Low	Low	High	Unclear	Low	Low	Low
Ryoo BY, 2020 (19)	Low	Low	Low	Low	Low	Low	Low

© 2022 Pala L et al. *JAMA Network Open.*

Larkin J, 2018 (20)	Low	Low	High	Low	Low	Low	Low
Schadendorf D; 2016 (21)	Low	Low	High	Unclear	Low	Low	Low
Sezer A, 2020; Sezer A, 2021 (22,23)	Low	Low	High	Unclear	Low	Low	Low
Cella D, 2016 (24)	Low	Low	High	Unclear	Low	Low	Low
Finn RS, 2019 (25)	Low	Low	High	Low	Low	Low	Low
Lewis K, 2020 (26)	Low	Low	Low	Low	Low	Low	Low
Bedke J, 2020 (27)	Low	Low	High	Low	Low	Low	Low
Adams S, 2020 (28)	Low	Low	Low	Unclear	Low	Low	Low
Mazieres J, 2019 (29)	Low	Low	Low	Low	Low	Low	Low
Garassino MC, 2020 (30)	Low	Low	Low	Low	Low	Low	Low
Kim HR, 2020 (31)	Low	Low	Low	Low	Low	Low	Low
Bamias A, 2020 (32)	Low	Low	Low	Low	Low	Low	Low
Goldman JW, 2020 (33)	Low	Low	High	Unclear	Low	Low	Low
Reck M, 2020 (34)	Low	Low	High	Low	Low	Low	Low
Mansfield AS, 2020 (35)	Low	Low	Low	Unclear	Low	Low	Low
Reck M, 2019 (36)	Low	Low	High	Low	Low	Low	Low
Cella D, 2019 (37)	Low	Low	High	Unclear	Low	Low	Low
Sherpereel A, 2020 (38)	Low	Low	High	Low	Low	Low	Low
Reck M, 2020 (39)	Low	Low	High	Low	Low	Low	Low

© 2022 Pala L et al. *JAMA Network Open.*

eTable 2. Assessment of quality of PROs reporting in RCTs included in the analysis.

Study	The PRO should be identified in the abstract as a primary or secondary outcome	The PRO hypothesis should be stated and relevant domains identified, if applicable	Evidence of PRO Instrument validity and reliability should be provided or cited if available	Statistical approaches for dealing with missing data are explicitly stated.	PRO-specific limitations and implications for generalizability and clinical practice should be discussed
Andre T, 2020 (4)	1	1	1	1	1
Van Cutsem E, 2019 (5)	1	1	1	0	0
Harrington KJ, 2020 (6)	1	1	1	1	1
Long GV, 2016 (7)	1	1	1	1	1
Reck M, 2018 (017) (8)	1	1	1	1	1
Reck M, 2018 (057) (9)	1	1	1	1	1
Barlesi F, 2018 (10)	1	1	1	1	1
Bordoni R, 2018 (11)	1	1	1	0	1
Hui R, 2019 (12)	1	1	1	1	1
Brahmer JR, 2017 (13)	1	1	1	1	1
Vaughn DJ, 2018 (14)	1	1	1	1	1
Powles T, 2017 (15)	0	1	1	0	0
Van Cutsem E, 2019 (16)	1	1	1	0	0
Harrington KJ, 2017, Ferris R, 2016 (17,18)	1	1	1	1	1
Ryoo BY, 2020 (19)	1	1	1	0	1
Larkin J, 2018 (20)	0	1	1	0	0

© 2022 Pala L et al. *JAMA Network Open.*

Schadendorf D; 2016 (21)	1	1	1	1	1
Sezer A, 2020; Sezer A, 2021 (22,23)	1	1	1	0	0
Cella D, 2016 (24)	1	1	1	1	1
Finn RS, 2019 (25)	0	1	1	0	0
Lewis K, 2020 (26)	1	1	1	0	0
Bedke J, 2020 (27)	1	1	1	0	0
Adams S, 2020 (28)	1	1	1	0	1
Mazieres J, 2019 (29)	1	1	1	1	1
Garassino MC, 2020 (30)	1	1	1	1	1
Kim HR, 2020 (31)	1	1	1	0	0
Bamias A, 2020 (32)	1	1	1	0	0
Goldman JW, 2020 (33)	1	1	1	1	1
Reck M, 2020 (34)	1	1	1	0	0
Mansfield AS, 2020 (35)	1	1	1	0	0
Reck M, 2019 (36)	1	1	1	0	1
Cella D, 2019 (37)	1	1	1	1	1
Sherpereel A, 2020 (38)	1	1	1	0	0
Reck M, 2020 (39)	1	1	1	0	0

© 2022 Pala L et al. *JAMA Network Open.*

eTable 3. Sensitivity analysis of TTD and GHS mean change excluding RCTs only available as congress abstracts.

	**TTD analysis**		**GHS mean change analysis**			
			**Week 12**		**Week 24**	
	**N comparisons**	**Pooled HR (95% CI)**	**N comparisons**	**Pooled difference in mean change (95% CI)**	**N comparisons**	**Pooled difference in mean change (95% CI)**
ICI monotherapy	9	0.78 (0.68, 0.90)	15	4.2 (2.2, 6.1)	13	5.8 (3.6, 8.0)
ICI + chemotherapy	2	0.90 (0.76, 1.07)	6	1.2 (-0.6, 3.1)	6	3.0 (-1.2, 7.1)
Other ICIs-containing combinations	3	0.71 (0.61, 0.81)	3	4.7 (-1.5, 10.9)	3	2.7 (-1.0, 6.3)

© 2022 Pala L et al. *JAMA Network Open.*

eReferences

1) Rohatgi A. WebPlotDigitizer. https://automeris.io/WebPlotDigitizer/ (2011)
2) Papadimitropoulou, K., Stijnen, T., Riley, R. D., Dekkers, O. M., & le Cessie, S. (2020). Meta-analysis of continuous outcomes: Using pseudo IPD created from aggregate data to adjust for baseline imbalance and assess treatment-by-baseline modification. Research synthesis methods, 11(6), 780-794.
3) Guyot P, Ades AE, Ouwens MJ, Welton NJ. Enhanced secondary analysis of survival data: reconstructing the data from published Kaplan-Meier survival curves. BMC Med Res Methodol. 2012; 12:9.
4) André T, Amonkar M, Norquist J, et al. Health-Related Quality of Life in Patients Treated With Pembrolizumab vs Chemotherapy as First-Line Treatment in Microsatellite Instability-High and/or Deficient Mismatch Repair Metastatic Colorectal Cancer: Phase 3 KEYNOTE-177 Study. Annals of Oncology (2020) 31 (suppl_4): S409-S461. 10.1016/annonc/annonc270
5) Van Cutsem E, Amonkar M, Fuchs CS, et al. Impact of pembrolizumab (pembro) versus paclitaxel on health-related quality of life (HRQoL) in patients with advanced gastric or gastroesophageal junction (GEJ) cancer that has progressed after firstline chemotherapy (KEYNOTE-061). Annals of Oncology (2019) 30 (suppl_5): v851-v934 10.1093/annonc/mdz247.117
6) Harrington KJ, Soulières D, Le Tourneau C, et al. Quality of Life With Pembrolizumab for Recurrent and/or Metastatic Head and Neck Squamous Cell Carcinoma: KEYNOTE-040. *J Natl Cancer Inst*. 2021;113(2):171-181
7) Long GV, Atkinson V, Ascierto PA, Robert C, Hassel JC, Rutkowski P, et al. Effect of nivolumab on health-related quality of life in patients with treatment-naïve advanced melanoma: results from the phase III CheckMate 066 study. Ann Oncol. 2016; 27(10): 1940¬1946
8) Reck M, Taylor F, Penrod J, et al. Impact of Nivolumab Versus Docetaxel on Health-Related Quality of Life and Symptoms in Patients With Advanced Squamous NonSmall Cell Lung Cancer: Results From the CheckMate 017 Study. J Thorac Oncol. 2018; 13(2): 194– 204
9) Reck M, Brahmer J, Bennett B, et al. Evaluation of health-related quality of life and symptoms in patients with advanced non-squamous non-small cell lung cancer treated with nivolumab or docetaxel in CheckMate 057. *Eur J Cancer*. 2018;102:23-30

© 2022 Pala L et al. *JAMA Network Open.*

10) Barlesi F, Garon EB, Kim DW, et al. Health-Related Quality of Life in KEYNOTE-010: a Phase II/III Study of Pembrolizumab Versus Docetaxel in Patients With Previously Treated Advanced, Programmed Death Ligand 1-Expressing NSCLC [published correction appears in J Thorac Oncol. 2019 Jul;14(7):1306]. *J Thorac Oncol*. 2019;14(5):793-801
11) Bordoni R, Ciardiello F, von Pawel J, Cortinovis D, Karagiannis T, Ballinger M, et al. Patient-reported outcomes in OAK: A phase III study of atezolizumab versus docetaxel in advanced non-small-cell lung cancer. Clin Lung Cancer 2018; 19(5): 441–449
12) Hui R, Özgüroğlu M, Villegas A, et al. Patient-reported outcomes with durvalumab after chemoradiotherapy in stage III, unresectable non-small-cell lung cancer (PACIFIC): a randomised, controlled, phase 3 study. *Lancet Oncol*. 2019;20(12):1670-1680
13) Brahmer JR, Rodriguez-Abreu D, Robinson AG, Hui R, Csôszi T, Fulôp A, et al. Health-related quality of-life results for pembrolizumab versus chemotherapy in advanced, PD-L1-positive NSCLC (KEYNOTE-024): a multicentre, international, randomised, open-label phase 3 trial. Lancet Oncol. 2017; 18 (12): 1600–160
14) Vaughn DJ, Bellmunt J, Fradet Y, Lee JL, Fong L, Vogelzang NJ, et al. Health-related quality of life analysis from keynote-45: A phase III study of pembrolizumab versus chemotherapy for previously treated advanced urothelial cancer. J Clin Oncol 2018; 16(1): 1579–158
15) Powles T, Durán I, van der Heijden MS, et al. Atezolizumab versus chemotherapy in patients with platinum-treated locally advanced or metastatic urothelial carcinoma (IMvigor211): a multicentre, open-label, phase 3 randomised controlled trial [published correction appears in Lancet. 2018 Oct 20;392(10156):1402]. *Lancet*. 2018;391(10122):748-757
16) Van Cutsem E, Valderrama A, Ban AJ, et al. Health-related quality of life (HRQoL) impact of pembrolizumab (P) versus chemotherapy (C) as first-line (1L) treatment in PD-L1–positive advanced gastric or gastroesophageal junction (G/GEJ) adenocarcinoma. Annals of Oncology (2019) 30 (suppl_5): v851-v934. 10.1093/annonc/mdz394
17) Harrington K, Ferris R, Blumenschein G, Colevas AD, Fayette J, Licitra L, et al. Nivolumab versus standard, single-agent therapy of investigator's choice in recurrent or metastatic squamous cell carcinoma of the head and neck (CheckMate 141): health-related quality-of-life results from a randomised phase 3 trial. Lancet Oncol. 2017; 18(8): 1104–1115

© 2022 Pala L et al. *JAMA Network Open.*

18) Ferris RL, Blumenschein G, Fayette J, Guigay J, Colevas AD, Licitra L, et al. Nivolumab for recurrent squamous-Cell carcinoma of the head and neck. N Engl J Med. 2016; 375: 1856–1867
19) Ryoo BY, Merle P, Kulkarni AS, et al. Health-related quality-of-life impact of pembrolizumab versus best supportive care in previously systemically treated patients with advanced hepatocellular carcinoma: KEYNOTE-240. *Cancer*. 2021;127(6):865-874
20) Larkin J, Minor D, D'Angelo S, Neyns B, Smylie M, Miller WH, et al. Overall survival in patients with advanced melanoma who received nivolumab versus investigator's choice chemotherapy in CheckMate 037: a randomized, controlled, open-label phase III Trial. J Clin Oncol. 2018; 36(4): 383–39
21) Schadendorf D, Dummer R, Hauschild A, Robert C, Hamid O, Daud A, et al. Health-related quality of life in the randomised KEYNOTE-002 study of pembrolizumab versus chemotherapy in patients with ipilimumab-refractory melanoma. Eur J cancer 2016; 67: 46–5
22) Sezer A, Kilickap S, Gümü M, et al. EMPOWER-Lung 1: Phase 3 First-line (1L) Cemiplimab Monotherapy vs Platinum-Doublet Chemotherapy (Chemo) in Advanced Non-Small Cell Lung Cancer (NSCLC) with Programmed Cell Death-Ligand 1 (PD-L1) ≥50%. Annals of Oncology (2020) 31 (suppl_4): S1142-S1215. 10.1016/annonc/annonc325
23) Sezer A, Kilickap S, Gümüş M, et al. Cemiplimab monotherapy for first-line treatment of advanced non-small-cell lung cancer with PD-L1 of at least 50%: a multicentre, open-label, global, phase 3, randomised, controlled trial. *Lancet*. 2021;397(10274):592-604
24) Cella D, Grünwald V, Nathan P, Doan J, Dastani H, Taylor F, et al. Quality of life in patients with advanced renal cell carcinoma given nivolumab versus everolimus in CheckMate 025: a randomised, open-label, phase 3 trial. Lancet Oncol. 2016; 17(7): 994–1003
25) Finn RS, Qin S, Ikeda M, et al. Atezolizumab plus Bevacizumab in Unresectable Hepatocellular Carcinoma. N Engl J Med. 2020;382(20):1894-1905
26) Lewis KD, Robert C, Ascierto P, et al.Patient-reported outcomes (PROs) from the phase III IMspire150 trial of atezolizumab (A) + cobimetinib (C) + vemurafenib (V) in patients (pts) with BRAFV600+melanoma. 10.1200/JCO.2020.38.15_suppl.10073
27) Health-Related Quality-of-Life Analysis From KEYNOTE-426: Pembrolizumab Plus Axitinib vs Sunitinib for Advanced Renal Cell Carcinoma. Presented by: Jens Bedke, MD, Professor and Chairman, Department of Urology Tuebingen, Tübingen, Germany Written by: Christopher J.D. Wallis, Urologic

Oncology Fellow, Vanderbilt University Medical Center, @WallisCJD on Twitter, at the Virtual 2020 EAU Annual Meeting #EAU20, July 17-19, 2020

28) Adams S, Diéras V, Barrios CH, et al. Patient-reported outcomes from the phase III IMpassion130 trial of atezolizumab plus nab-paclitaxel in metastatic triple-negative breast cancer. *Ann Oncol*. 2020;31(5):582-589
29) Mazieres J, Kowalski D, Luft A, et al. Health-Related Quality of Life With Carboplatin-Paclitaxel or nab-Paclitaxel With or Without Pembrolizumab in Patients With Metastatic Squamous Non-Small-Cell Lung Cancer. *J Clin Oncol*. 2020;38(3):271-280
30) Garassino MC, Gadgeel S, Esteban E, et al. Patient-reported outcomes following pembrolizumab or placebo plus pemetrexed and platinum in patients with previously untreated, metastatic, non-squamous non-small-cell lung cancer (KEYNOTE-189): a multicentre, double-blind, randomised, placebo-controlled, phase 3 trial. *Lancet Oncol*. 2020;21(3):387-397
31) Kim HR, Axa MM, Navarro A, et al. Health-related quality of life (HRQoL) in KEYNOTE-604: Pembrolizumab (pembro) or placebo added to etoposide and platinum (EP) as first-line therapy for ES-SCLC. Annals of Oncology (2020) 31(suppl_ 4):S1033-S1034. 10.1016/j.annonc.2020.08.1544
32) Bamias A, De Santis M, Arranz JA, et al. Patient-reported outcomes (PROs) from IMvigor130: A global, randomised, partially blinded phase III study of atezolizumab (atezo) + platinum-based chemotherapy (PBC) vs placebo (PBO) + PBC in previously untreated locally advanced or metastatic urothelial carcinoma (mUC). Annals of Oncology (2020) 31 (suppl_4): S550-S550. 10.1016/annonc/annonc274
33) Goldman JW, Garassino MC, Chen Y, et al. Patient-reported outcomes with first-line durvalumab plus platinum-etoposide versus platinum-etoposide in extensive-stage small-cell lung cancer (CASPIAN): a randomized, controlled, open-label, phase III study. Lung Cancer. 2020;149:46-52
34) Reck M, Wehler T, Orlandi F, et al. Safety and Patient-Reported Outcomes of Atezolizumab Plus Chemotherapy With or Without Bevacizumab Versus Bevacizumab Plus Chemotherapy in Non-Small-Cell Lung Cancer. J Clin Oncol. 2020;38(22):2530-2542.
35) Mansfield AS, Każarnowicz A, Karaseva N, et al. Safety and patient-reported outcomes of atezolizumab, carboplatin, and etoposide in extensive-stage small-cell lung cancer (IMpower133): a randomized phase I/III trial. Ann Oncol. 2020;31(2):310-317
36) Reck M, Schenker M, Lee KH, et al. Nivolumab plus ipilimumab versus chemotherapy as first-line treatment in advanced non-small-cell lung cancer

© 2022 Pala L et al. *JAMA Network Open.*

with high tumour mutational burden: patient-reported outcomes results from the randomised, open-label, phase III CheckMate 227 trial. Eur J Cancer. 2019;116:137-147

37) Cella D, Grünwald V, Escudier B, et al. Patient-reported outcomes of patients with advanced renal cell carcinoma treated with nivolumab plus ipilimumab versus sunitinib (CheckMate 214): a randomised, phase 3 trial [published correction appears in Lancet Oncol. 2019 Jun;20(6):e293]. Lancet Oncol. 2019;20(2):297-310
38) Scherpereel A, Antonia S, Bautista Y, et al. First-line nivolumab (NIVO) plus ipilimumab (IPI) versus chemotherapy (chemo) for the treatment of unresectable malignant pleural mesothelioma (MPM): Patient-reported outcomes (PROs) from CheckMate 743. Annals of Oncology (2020) 31 (suppl_7): S1441-S1451. 10.1016/annonc/annonc392
39) Reck M, Ciuleanu TE, Cobo M, et al.First-line nivolumab + ipilimumab combined with 2 cycles of platinum-based chemotherapy versus 4 cycles of chemotherapy in advanced non-small cell lung cancer: Patient-reported outcomes from CheckMate 9LA. Annals of Oncology (2020) 31 (suppl_4): S1142-S1215. 10.1016/annonc/annonc325
